# Cumulative Trauma and Trauma Symptoms: A Three-Way Interaction

**DOI:** 10.3390/bs13070576

**Published:** 2023-07-11

**Authors:** Fang Xue, Han Na Suh, Kenneth G. Rice, Jeffrey S. Ashby

**Affiliations:** Department of Counseling and Psychological Services, Georgia State University, Atlanta, GA 30303, USA; fxue2@gsu.edu (F.X.); hsuh@gsu.edu (H.N.S.); kgr1@gsu.edu (K.G.R.)

**Keywords:** cumulative trauma, PTSD symptoms, perceived social support, self-compassion

## Abstract

The purpose of this study was to test if perceived social support and self-compassion will interact to reduce the magnitude of the bivariate relationship (buffering effect) between cumulative trauma and trauma symptoms after controlling for gender and age among college students. As part of a broader research project conducted between 2018 and 2019, we collected data via online surveys from a sample of 551 undergraduate students at a public university in the southern region of the US. After data cleaning, the study included 538 participants (representing 97.6% of the original dataset), ensuring a diverse representation across various ethnicities and genders. The three-way interaction model accounted for 38.61% of the variance in PTSD symptoms. In detail, with high levels of perceived social support, there was a significant difference in the buffering effects of perceived social support on the trauma–PTSD association between high and low self-compassion. Conversely, at high levels of self-compassion, perceived social support did not significantly influence the buffering effect of self-compassion. This study underscores the critical role of self-compassion in enhancing the protective effect of high-level perceived social support against PTSD symptoms following cumulative trauma.

## 1. Introduction

The prevalence of exposure to potentially traumatic events (PTEs) among American college students is surprisingly as high as 50% to 76% [1,2,3,4]. College students endorse various types of PTEs, including accidents (e.g., car accidents, construction accidents, life-threatening accidents), natural disasters, warfare, sudden unexpected death, life-threatening illness, physical violence, and unwanted sex [4]. The COVID-19 outbreak has further amplified the intensity and frequency of traumatic experiences endured by minority communities [5,6]. Several studies have found a significant relationship between exposure to PTEs and risks for psychopathology, including substance abuse [7], depression and anxiety [8], and post-traumatic stress disorder (PTSD) [9]. In addition, college students who reported the experience of PTEs tend to exhibit poorer physical health and academic performance [10]. 

Some individuals may have experienced multiple types of traumatic experiences, and researchers have termed this cumulative trauma. Cumulative trauma, defined as “the effects of all the trauma types the person endured across time” [11] (p. 181), may result in complex PTSD and yield more severe symptoms of psychopathology than single-event traumas [12,13]. This dose effect of PTEs on mental health has also been investigated in nonclinical samples. For example, a study of first-year undergraduates at an urban, public university in the United States’ southern region found that the intensity of trauma-related stress increased with the number of PTEs experienced by students [8]. Previous research has provided evidence of the adverse effects of cumulative trauma on college students, highlighting the need to explore the mechanisms by which protective factors can act as buffers against psychopathology.

### 1.1. Theoretical Framework: The Conservation of Resources Theory

Hobfoll’s conservation of resources (COR) theory, developed in 1988, provides a theoretical framework to understand how traumatic experiences affect survivors and how survivors recover and has been supported by empirical work on stress and trauma. COR theory is centered on individuals striving to obtain and preserve valuable resources to ensure their survival and well-being [14]. Hobfoll divided resources into three categories: primary resources are those most closely and directly related to survival, such as water, security, and food; secondary resources indirectly help people access primary resources, such as social support and optimism; finally, tertiary resources are culturally constructed resources that are associated with primary and secondary resources, such as social status [15]. Stressors, which are events in the environment that threaten or prevent people from accessing the resources they are maintaining, can become even more severe when traumatic events occur, leading to the loss of multiple resources or even threatening people’s lives [15]. When resources are depleted or continuously lost, psychopathological symptoms may be developed [15].

COR theory also sheds light on the unique and more profound impacts of cumulative trauma compared to single traumatic incidents. Hobfoll et al. [15] proposed that individuals possessing fewer initial resources demonstrate diminished resilience in response to traumatic events. The second corollary of COR theory, which states that “initial loss begets future loss” [15] (p. 68), explains that using resources to navigate traumatic experiences often leads to resource depletion, resulting in increased vulnerability to subsequent traumas as the initial resource pool contracts with every experienced trauma [15]. For example, when a college student in a car accident loses physical integrity as a vital resource and leans on family support to cope with the trauma, subsequent loss of their family due to another trauma further diminishes his or her resource pool, leaving the student without both the initial physical integrity and the recently relied-upon family support. This cumulative trauma would result in an escalation of psychological distress due to the severe depletion of coping resources.

### 1.2. Perceived Social Support and Self Compassion as Resources

A number of authors have defined perceived social support as the perceived accessibility of material, psychological, and holistic support from friends, family, and others, particularly when individuals need these resources [16,17]. Conservation of resources (COR) theory proposes that perceived social support operates as a secondary resource, assisting individuals in accessing their primary resources [15]. Beyond the direct provision of primary resources through social support, research suggests that individuals who perceive higher levels of social support are more likely to employ effective coping strategies and reduce risky behaviors [18,19], highlighting how perceived social support can enhance the effective utilization of primary resources. Empirical evidence has indicated that perceived social support can significantly buffer cumulative trauma’s negative impact on college students’ psychological well-being [20]. Similar findings regarding the protective role of perceived social support have also been observed in more vulnerable groups, such as the research conducted by Schumm et al. [21], which demonstrated that high levels of perceived social support can mitigate the cumulative impact of interpersonal traumas among women residing in inner-city communities.

Self-compassion, which involves extending kindness and empathy towards oneself during stressful circumstances, can be categorized as a secondary resource according to Hobfoll et al.’s classification of resource types [15]. The concept comprises three interconnected components: (1) self-kindness, characterized by a caring and understanding attitude towards oneself devoid of judgment or criticism, (2) common humanity, acknowledging that personal suffering is part of the universal human experience, and (3) mindfulness, the conscious awareness of one’s present moment experience [22,23]. Several studies have found that, similar to perceived social support, self-compassion can serve as a protective factor against the effects of traumatic events [24,25,26], facilitating individuals who have experienced potentially traumatic events (PTEs) to regain control, security, and a sense of predictability in their lives and environments [27,28]. Notably, Shebuski et al. [29] found that self-compassion buffered the relationship between trauma exposure and psychological distress among college students.

### 1.3. The Interplay between Perceived Social Support and Self Compassion

Hobfoll et al. [30] identify resources that develop and maintain interdependently as resource caravans. Hobfoll [30] provides an example of the resource caravan of self-esteem, self-efficacy, and optimism as interdependent resources connected developmentally. Hobfoll et al. [15] contend that individuals possessing resource caravans regenerate resources more efficiently after experiencing trauma, reducing their risk of developing psychopathological symptoms. Conversely, individuals lacking resource caravans may struggle to efficiently regenerate resources, rendering them more susceptible to loss spiral and the emergence of psychopathological symptoms [15].

Perceived social support and self-compassion, two distinct but related resources, align with this concept of resource caravans. Research suggests that self-compassion may enhance perceived social support by fostering increased interpersonal connectedness [31,32]. Conversely, perceived social support has been identified as a crucial factor in developing self-compassion. Neff and McGehee [33] suggest that individuals receiving more significant support and affirmation from their environment are more likely to adopt a self-compassionate attitude than those receiving less support. The synergistic development of self-compassion and perceived social support underscores a complex interplay that may significantly impact the association between cumulative trauma and PTSD symptoms.

### 1.4. The Present Study

While both COR theory and empirical studies suggest that perceived social support and self-compassion may function collaboratively to buffer the impact of cumulative trauma on PTSD symptoms, few investigations have investigated the interactive dynamics of these two protective factors considering the impacts of cumulative trauma. The current study aims to examine whether and how perceived social support and self-compassion interact to moderate the association between cumulative trauma and PTSD symptoms among college students while controlling for age and gender. To address our question, we used a cross-sectional design to collect data and multiple regression to analyze data. Details on data collection measures and analysis processes follow.

## 2. Materials and Methods

### 2.1. Participants and Procedure

After receiving IRB approval, we recruited participants via an online SONA system from a southeastern university. Participants were provided credits to fulfill their psychology classes’ research requirements. Through the SONA system, participants received a link to Qualtrics, where they were then presented with informed consent, followed by scales. The data set was collected from 2018 to 2019.

The final sample included 538 undergraduate participants from a large southeastern public university. Of the 538 participants, 54.8% identified as cisgender female, while 45.2% as cisgender male. Our sample was racially diverse, with 40.1% identifying as Black/African American, 26.4% as White/Caucasian, 17.7% as Asian/Asian American, 8.7% as Latino(a)/Hispanic, 5.6% as multiracial, and 1.5% as identifying under “other” racial categories. Four participants did not provide racial information. A total of 51.7% of the sample identified as Christian, making it the most common religious affiliation. This was followed by 14.2% who declared themselves atheists, 7.7% as Muslim, and 7.5% as agnostic. Additionally, 3.2% reported their religion as Buddhism, 2.9% as Hinduism, and 1.8% as Judaism. Another 9.8% categorized their beliefs as ”other”. A small subset, 1.2% (or seven students), chose not to provide information on their religious beliefs. Nearly two-thirds of respondents (65.6%) reported an annual income under USD 25,000. The age range spanned from 18 to 40 years (M = 23.10, SD =3.97).

### 2.2. Measures

#### 2.2.1. Trauma Symptom Checklist-40 (TSC-40)

The TSC-40 is a self-report, 40-item scale designed to assess the psychological and physiological symptoms caused by traumatic experiences [34]. Although the scale is widely used to measure the traumatic impact of chronic child abuse, it has also been used to measure post-traumatic symptomatology associated with other types of traumatic events [34]. The scale delves into six categories of trauma symptoms, such as anxiety, depression, and sleep disturbance [34]. In the present study, we employed the overall score generated by the TSC-40 to represent the comprehensive impact of cumulative trauma. Participants reported how often they have experienced 40 traumatic symptoms in the past two months, with a score of 0 being “never” and 3 being “often.” Elliott and Briere [34] provided evidence for the scale’s internal reliability (α = 0.90) among a sample of professional women in the United States. Rizeq et al. [35] found high internal reliability (Ω = 0.93) and strong invariance across individuals with different trauma histories (i.e., with or without trauma and varying numbers of types of trauma experienced) within a sample of undergraduate students in Canada. The authors concluded that the scores obtained from the TSC-40 showed adequate convergent validity in assessing complex trauma sequelae across multiple types of trauma in their study sample [35]. The omega reliability estimate for this measure in our sample was 0.95.

#### 2.2.2. The Trauma History Screen (THS)

The Traumatic History Screen (THS) is a self-report instrument designed to assess individuals’ lifetime exposure to trauma, encompassing high magnitude stressors (HMS) and events linked to significant and persisting post-traumatic distress (PPD) [36]. The scale comprises a dichotomous categorical variable (participants respond with yes/no regarding exposure to each trauma category), a continuous variable (participants indicate the frequency of experiencing each trauma type), and detailed information pertaining to PPD events (e.g., the duration of the endorsed traumatic event) [36]. Example traumatic events include “Forced or made to have sexual contact—as a child” and “A hurricane, flood, earthquake, tornado, or fire” [36]. In this study, cumulative trauma is defined as the number of different types of trauma experienced. Therefore, we concentrated solely on the types of trauma experienced by calculating the total number of traumas that the participant acknowledged with a “yes” response. Carlson et al. [36] reported that the THS exhibited high test–retest reliability (r = 0.87) among college students.

#### 2.2.3. The Self-Compassion Scale (SCS)

The SCS is a 26-item scale to assess participants’ self-compassion [37]. The questionnaire consists of six subscales, including Self-Kindness, Self-Judgment, Common Humanity, Isolation, Mindfulness, and Over-Identified [37]. Neff [37] indicated that self-compassion is an overarching factor reflected by the scores on the six subscales combined, and each pair of negative and positive scales reflects an essential component of self-compassion. Consistent with previous studies [32,38], this study used the overall score to assess self-compassion in the proposed model. Sample items in the SCS include: “I’m disapproving and judgmental about my own flaws and inadequacies” and “I try to be loving towards myself when I’m feeling emotional pain” [37]. Participants were asked to indicate how often they behave in the stated manner on a scale of 0 (almost never) and 5 (almost always). Neff [37] demonstrated that the overall score exhibits high test–retest reliability (r = 0.93) among college students. Additionally, Castilho et al. [39] revealed that the overall scale maintains stable internal consistency, with coefficient alphas ranging from 0.92 to 0.94 for the total score in both clinical and nonclinical settings. The omega reliability estimate for this measure in our sample was 0.91.

#### 2.2.4. The Multidimensional Scale of Perceived Social Support (MSPSS)

The 12-item MSPSS scale was used to assess participants’ perceived social support [40]. The scale consists of three subscales, asking participants about perceived social support from their significant others, families, and friends [40]. As widely used in other studies [22,41], this study used the total score to measure participants’ overall level of perceived social support. Sample items include: “My family really tries to help me” and “I can count on my friends when things go wrong”. Participants were asked to respond to each item on a scale of 1 (very strongly disagree) to 7 (very strongly agree). Zimet et al. [40] found that the overall scale demonstrates good internal reliability, with a range of 0.84 to 0.92 across populations. The omega reliability estimate for this measure in our sample was 0.93.

### 2.3. Data Preparatory Strategy

We removed two observations for the data preparation and analysis because gender was included as a covariate. The two observations represent only 0.4% of the sample: one self-identified as transgender, and another selected the “Other” option. We decided to remove them because they may not accurately represent the subpopulation and could introduce bias. We also removed 11 observations due to their classification as outliers, given their age standardized scores exceeded a threshold of 3.29 [42]. As our target population is undergraduate students, we hope to ensure our dataset reflected the characteristics of this demographic. Therefore, we chose to eliminate these outliers to maintain a representative sample.

A preliminary analysis revealed that the trauma symptoms total score had the highest percentage of missing data at 21.4%, followed by the self-compassion total score at 15.8%, perceived social support total score at 2.6%, age at 1.5%, and gender at 0.6%. The two variables with the highest percentage of missing data correspond to scales with the most items: the Trauma Symptoms Checklist, including 40 items, and the Self-Compassion Scale, requiring responses from 26 items. At the item level, the percentage of missing data for the Trauma Symptoms Checklist ranged from 0.9% to 2.4%. Similarly, for the Self-Compassion Scale, the item-level percentage of missing data varied from 1.1% to 1.9%. We proceeded to analyze the missingness at the item level because the pattern of missingness is more likely to emerge at this level when sample sizes exceed 100 [43]. We followed the suggested steps in Schlomer et al. [44] and ran Little’s test [45] via SPSS version 27 to analyze the pattern of missingness. The results were statistically insignificant (χ^2^ = 8968.704, df = 9303, *p* = 0.993), suggesting the missing data are likely to be completely at random.

We applied the expectation maximization (EM) algorithm to replace missing values at the item level and then recalculated the scale total scores using the imputed values [43]. We decided to use the EM algorithm because (1) datasets with data missing completely at random fit the assumption of EM algorithm, and (2) the approach provides unbiased parameters [46].

## 3. Results

### 3.1. Descriptive Analyses

Table 1 provides descriptive statistics, including means, standard deviations for continuous variables, and correlation coefficients for all variables of interest. The significant correlations were in the expected direction, and the effect sizes were weak to moderate.

### 3.2. Moderator Analyses

Following the guidelines described by Pallant [47], we ensured that the assumptions requisite for multiple regression were met. The research question of our study was whether perceived social support and self-compassion interact to buffer the association between cumulative trauma and trauma symptoms among college students after controlling for age and gender. We used SPSS PROCESS macros version 4.2 to explore the question, employed a bootstrapping procedure with 5000 samples and mean-centering the continuous dependent variables [48].

Gender and age were included as covariates because previous studies evidenced their influence on trauma experience and their interactions impacting PTSD symptoms [49,50,51,52]. We coded men as “1” and women as “0”. Perceived social support and self-compassion were tested as moderators, and the focal association involved the interaction between cumulative trauma, perceived social support, and self-compassion in relation to PTSD symptoms.

**Figure 1:**
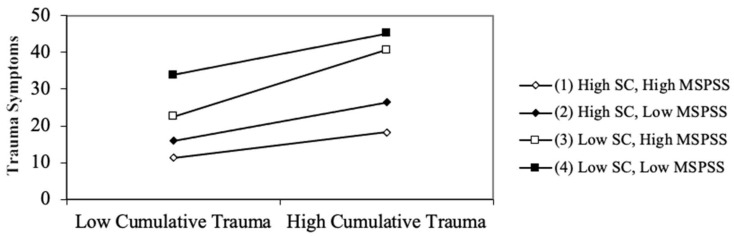
Three-way interaction of cumulative trauma, perceived social support, and self-compassion on PTSD symptoms.

The full model accounted for 38.61% of the variance in the Trauma Symptoms Checklist-40 scores (TSC-40), R2 = 0.39, F (9, 528) = 36.90, *p* < 0.001. As shown in Table 2, the three-way interaction was statistically significant, indicating that the impact of cumulative trauma on trauma symptoms is contingent upon the levels of self-compassion and perceived social support, even when controlling for age and gender. We employed simple slopes, slope differences, and visual representations to elucidate this complex three-way interaction. We generated four simple slopes, representing the conditions of (1) low perceived social support (MSPSS) and low self-compassion (SC), (2) high MSPSS and low SC, (3) low MSPSS and high SC, and (4) high MSPSS and high SC. Both “low” and “high” levels are defined as one standard deviation below and above the mean, respectively. We then compared these slopes and displayed the significant slope difference result in Figure 1.

In general, there was a positive and significant association between cumulative trauma and PTSD symptoms, meaning that as the number of different types of trauma experienced increased, the TSC-40 scores rose. The simple slope results revealed statistically significant effects of CT on PTSD symptoms at all levels of MSPSS and SC (*p* < 0.05). Specifically, when the MSPSS level was low, the simple slopes for the association between CT and TSC-40 were B = 2.51 (SE = 0.55) at the low SC level, and B = 2.35 (SE = 0.74) at the high SC level. When the MSPSS level was high, the simple slopes for the association between CT and TSC-40 were B = 4.06 (SE = 0.80) at the low SC level and B = 1.55 (SE = 0.55) at the high SC level.

The slopes were then compared for differences. The only statistically significant slope difference found was between the high MSPSS–low SC and the high MSPSS–high SC conditions. In particular, the slope was steeper (by 2.51 units) for the high MSPSS–low SC condition compared to the high MSPSS–high SC condition, and this difference was statistically significant, t(536) = −2.63, *p* = 0.009. The result suggests that when the MSPSS level was high, the impact of CT on PTSD symptoms was significantly more substantial when the SC level was low as opposed to when it was high. All other comparisons demonstrated *p*-values exceeding the 0.05 threshold, and the comparison between low SC–low MSPSS and low SC–high MSPSS yielded the lowest *p*-value of 0.097 among them.

## 4. Discussion

The results of this study suggest that the interaction between self-compassion and perceived social support significantly modify the magnitude of the association between cumulative trauma and PTSD symptoms. Interestingly, when perceived social support was at a high level, self-compassion significantly modified perceived social support’s buffering effect. This manifests as a high level of self-compassion, further attenuating the association between cumulative trauma and PTSD symptoms. However, when individuals exhibit a high level of self-compassion, the level of perceived social support—regardless of being high or low—does not significantly affect the buffering effect of self-compassion. This nuanced distinction suggests that the availability of specific resources—perceived social support or self-compassion—can influence an individual’s resilience against cumulative trauma.

The finding that self-compassion can significantly modify the buffering effect of perceived social support aligns with the second principle of COR theory, emphasizing the importance of both possessing and effectively utilizing resources [15]. These results suggest that, when individuals possess abundant external resources (perceived social support), the critical factor in managing traumatic stress becomes whether they have the internal resources (self-compassion) to capitalize on these external resources effectively. Past research has shown that individuals with lower self-compassion tend to exhibit defensiveness or denial rather than acceptance of reality, compared to individuals with higher self-compassion [53,54]. In the context of our study, it is plausible that individuals with lower self-compassion may underutilize or avoid external resources like social support due to their denial of problems, thus diminishing the buffering effect of perceived social support.

In contrast, when individuals have higher levels of self-compassion, perceived social support does not significantly modify the buffering effects of self-compassion. One explanation of this finding is that individuals with higher levels of self-compassion are proficient in harnessing diverse external resources to confront trauma, which extends beyond social support. For example, Bonanno and Galea [55] found that material resources such as income, energy resources like physical health, and social resources such as social support were all significant predictors of psychological resilience following a natural disaster. Therefore, the specific role of social support, being one facet of these external resources, may not stand out as prominently in its beneficial impacts on resilience.

An alternative interpretation might be that individuals with high levels of self-compassion may not need to utilize many external resources, given that they already possess adequate internal resources to fortify themselves against trauma. Maheux and Price [22] have proposed that self-compassion could be a pathway through which perceived social support alleviates psychopathological symptoms. They have further suggested that their findings might lend preliminary support to a theoretical mechanism positing that one function of perceived social support in the context is to transform into self-compassion. In other words, internal resources, rather than external ones, may have a more direct correlation with psychopathological symptoms.

When interpreting the results of our study, it is crucial to highlight notable demographic features. One prominent characteristic of our sample population, with a mean age of 23, is that it skews older than the typical age-range of traditional college students, generally between 18 and 22 years [56]. This age difference suggests potential variations in our sample’s life experiences. For instance, nontraditional college students in our sample may carry financial responsibilities for their families or navigate the challenges of balancing full-time employment with their enrollment [57]. These unique life experiences have the potential to introduce diverse coping mechanisms into their lives. Furthermore, our sample exhibited a higher average cumulative trauma count of 3.01. In comparison, the Briere et al. study [58] reported an average of 2.2 trauma types within a general population sample, while Vrana and Lauterbach [59] found that college students experienced a mean number of traumatic events ranging from 2.52 to 2.98. The slightly elevated cumulative trauma load within our sample may impact how the moderating effects of our model operate.

### 4.1. Implications

#### 4.1.1. Future Research

To enhance the richness of numerical findings, we advocate using qualitative or mixed-method research approaches in future studies. Specifically, we recommend utilizing Online Photovoice (OPV), an innovative and effective qualitative research method, for related explorations. OPV provides a platform for participants to use imagery to express their strengths and concerns, fostering discussion as they comment on each other’s submissions [60]. Furthermore, it opens an interactive exhibition for policymakers and other interested parties [60]. We suggest that, within the context of trauma studies, OPV could yield therapeutic effects by bolstering self-compassion and empowerment, giving voice to cumulative trauma survivors.

Despite its relative neglect in the PTSD literature, Kira et al. [61] proposed that collective identity traumas, including discrimination and oppression, constitute a significant form of trauma. They argued that these experiences are particularly impactful due to their ongoing nature and potential to shape individual identity development. This notion aligns with Williams et al.’s findings [62] that all forms of oppression carry a traumatic impact. Given these insights, we believe it is essential to broaden the “cumulative trauma” concept used in research studies. By incorporating scores from scales that assess collective identity trauma, such as the Racial Microaggressions Scale [63] and the General Ethnic Discrimination Scale [64], we could achieve a more inclusive and comprehensive representation of trauma.

#### 4.1.2. Mental and Public Health Professionals

The first recommendation for mental health professionals is to prioritize the cultivation of self-compassion, particularly in scenarios of constrained resources. Self-compassion, as an internal resource, seems to play a crucial role in determining whether an individual can effectively utilize external resources. Also, self-compassion is more readily available than perceived social support, given that it is an attribute one can nurture and rely on internally. This inherent accessibility makes self-compassion an invaluable tool, providing a source of resilience that individuals can utilize regardless of their external circumstances. A meta-analysis by Reilly and Stuyvenberg [65] demonstrated a moderate overall effect of loving-kindness meditations on self-compassion among adults. Their findings further suggest that the delivery mode of these meditations does not influence their beneficial impact. Therefore, universities could offer online loving-kindness meditation recordings as a resource for students who may require assistance but cannot readily access it.

When resources are plentiful, it is recommended that mental health professionals initiate programs that aim to nurture both perceived social support and self-compassion. Although it was not the focal point in this three-way interaction outcome, consistent with conservation of resources theory, it was evident that individuals with high levels of both self-compassion and perceived social support displayed fewer PTSD symptoms, as illustrated in Figure 1. Consequently, implementing programs to bolster both resources, such as group-based mindfulness-based cognitive therapy (MBCT) or group-based acceptance and commitment therapy (ACT), could optimize the unique benefits of both external and internal resources [66,67].

### 4.2. Limitations

The major limitation was using a retrospective self-report scale to measure participants’ cumulative trauma. Retrospective self-reporting is often used to collect data on traumatic events, and empirical studies have demonstrated its reliability and validity [68,69]. However, recalling exactly what happened in the past is difficult, and trauma-related events can exaggerate or suppress memories because of the unpleasant emotions they evoke, making accurate recall even difficult [70]. In addition, the data used in this study are cross-sectional and therefore do not suggest any causal relationships. Lastly, the TSC-40 questionnaire may not fully capture the effects of self-compassion and perceived social support. Winders et al. [30] suggested that self-compassion is negatively associated with distorted cognitions that often arise following traumatic events, such as fear, shame, and self-blame. However, the TSC-40, employed in this study, does not have a dedicated subscale for distorted cognitions and might not provide a comprehensive assessment of these aspects of trauma symptoms. Similarly, Simon et al. [71] found that perceived social support was significantly associated with ICD-11 Complex PTSD symptoms and DSM-5 criterion D but not with ICD-11 PTSD symptoms or DSM-5 criteria B, C, and E. In other words, perceived social support may be more effective in regulating emotion dysregulation, negative self-perceptions, and interpersonal problems for individuals who experienced trauma. However, the TSC-40 might not have been comprehensive enough to capture these specific aspects of trauma symptoms. Future studies might combine the use of the TSC-40 with the other measures like the Post-traumatic Cognitions Inventory (PTCI) [72] to gain a more exhaustive understanding of the relationships between self-compassion, social support, distorted cognitions, and trauma symptoms.

## 5. Conclusions

In summary, our study highlights the pivotal role of self-compassion in amplifying the protective effects of high-level perceived social support against PTSD symptoms. Drawing from Hobfoll’s conservation of resources theory, our findings illuminate the importance of internal resources for trauma survivors, enabling them to utilize external resources more efficiently. This suggests that fostering high self-compassion could be a crucial strategy in trauma interventions.

To better assist survivors of cumulative trauma, we urge the development of more comprehensive trauma-informed care strategies emphasizing the interplay between internal and external resources in trauma recovery. In future research, we recommend a more comprehensive exploration of coping mechanisms, considering other potential covariates and variables. Lastly, we hope future studies adopt innovative study designs and measurement tools to address the limitations identified in our study.

## Figures and Tables

**Table 1:** Descriptive statistics and bivariate correlation of research variables.

	M(SD)	1	2	3	4	5	6
1. Traumatic symptoms	27.37 (20.77)	1					
2. Cumulative Trauma	3.01 (2.23)	0.35 *	1				
3. Self-Compassion	3.15 (0.64)	−0.51 *	−0.15 *	1			
4. Perceived Social Support	5.17 (1.31)	−0.29 *	−0.04	0.28 *	1		
5. Age	23.10 (3.97)	0.04	0.20 *	0.07	−0.03	1	
6. Gender		−0.20 *	−0.15 *	0.11 *	0.02	−0.04	1

Significant correlations are marked with “*” (*p* < 0.01, two-tailed).

**Table 2:** Regression analysis for predictors of PTSD symptoms, controlling for age and gender.

Predictor	*B*	*SE*	*p*
Gender	−4.57	1.45	0.002
Age	0.11	0.18	0.557
Cumulative Trauma (CT)	2.62	4.35	0.000
Perceived Social Support (MSPSS)	−2.73	0.58	0.000
Self-Compassion (SC)	−13.76	1.20	0.000
CT × SC	−1.05	0.55	0.058
CT × MSPSS	0.14	0.27	0.596
SC × MSPSS	0.44	0.81	0.590
CT × SC × MSPSS	−0.70	0.34	0.040

## Data Availability

The data presented in this study are available on request from the corresponding author. The data are not publicly available due to privacy.
